# Lumbar spinal canal MRI diameter is smaller in herniated disc cauda equina syndrome patients

**DOI:** 10.1371/journal.pone.0186148

**Published:** 2017-10-12

**Authors:** Nina S. Korse, Mark C. Kruit, Wilco C. Peul, Carmen L. A. Vleggeert-Lankamp

**Affiliations:** 1 Department of Neurosurgery, Leiden University Medical Centre, Leiden, the Netherlands; 2 Department of Radiology, Leiden University Medical Centre, Leiden, the Netherlands; 3 Department of Neurosurgery, Haaglanden Medical Centre, The Hague, the Netherlands; Rush University Medical Center, UNITED STATES

## Abstract

**Introduction:**

Correlation between magnetic resonance imaging (MRI) and clinical features in cauda equina syndrome (CES) is unknown; nor is known whether there are differences in MRI spinal canal size between lumbar herniated disc patients with CES versus lumbar herniated discs patients without CES, operated for sciatica. The aims of this study are 1) evaluating the association of MRI features with clinical presentation and outcome of CES and 2) comparing lumbar spinal canal diameters of lumbar herniated disc patients with CES versus lumbar herniated disc patients without CES, operated because of sciatica.

**Methods:**

MRIs of CES patients were assessed for the following features: level of disc lesion, type (uni- or bilateral) and severity of caudal compression. Pre- and postoperative clinical features (micturition dysfunction, defecation dysfunction, altered sensation of the saddle area) were retrieved from the medical files. In addition, anteroposterior (AP) lumbar spinal canal diameters of CES patients were measured at MRI. AP diameters of lumbar herniated disc patients without CES, operated for sciatica, were measured for comparison.

**Results:**

48 CES patients were included. At MRI, bilateral compression was seen in 82%; complete caudal compression in 29%. MRI features were not associated with clinical presentation nor outcome. AP diameter was measured for 26 CES patients and for 31 lumbar herniated disc patients without CES, operated for sciatica. Comparison displayed a significant smaller AP diameter of the lumbar spinal canal in CES patients (largest p = 0.002). Compared to average diameters in literature, diameters of CES patients were significantly more often below average than that of the sciatica patients (largest p = 0.021).

**Conclusion:**

This is the first study demonstrating differences in lumbar spinal canal size between lumbar herniated disc patients with CES and lumbar herniated disc patients without CES, operated for sciatica. This finding might imply that lumbar herniated disc patients with a relative small lumbar spinal canal might need to be approached differently in managing complaints of herniated disc. Since the number of studied patients is relatively small, further research should be conducted before clinical consequences are considered.

## Introduction

Cauda equina syndrome (CES) is a rare neurological complication caused by compression of the nerve roots of the cauda equina. CES is defined by presence of one or more of the following symptoms: 1) bladder and/or bowel dysfunction, 2) reduced sensation of the saddle area and/or 3) sexual dysfunction, with possible neurologic deficit in the lower limb [1]. CES can be instigated by several processes, such as lumbar herniated disc, tumour, infection, stenosis or hematoma, with lumbar herniated disc being the most common cause described in literature (45%) [1]; CES provoked by other pathology is beyond the scope of this article. CES is regarded as an emergency indication for surgery, and the value of early surgery has been supported by–among others–the well-known meta-analysis of Ahn et al. (2000), demonstrating that CES patients surgically decompressed within 48 hours have a significant better outcome of sensory, motor, urinary and rectal function compared to those being operated after 48 hours [2].

The diagnosis of CES is based on a combination of clinical and imaging features. Interpretation of clinical features alone is difficult due to the great inter patient variation of symptoms. Magnetic resonance imaging (MRI) of the lumbar spine is the current modality of choice in any suspected case of CES to confirm diagnosis and to identify the causative agent and level of caudal compression [3].

Only 1–10% of patients with a known lumbar herniated disc develop CES [4–6], and it is not (yet) possible to predict which lumbar herniated disc patients will develop CES. By reasoning, a factor such as the (premorbid) lumbar spinal canal size might play a part in the development of clinically evident caudal compression in lumbar herniated disc patients. Exploring imaging characteristics that may herald a higher risk for CES—such as spinal canal size—might create a unique opportunity for early surgery in known lumbar herniated disc patients not yet affected by CES. Prevention is better than cure especially in case of CES, due to rather disappointing postoperative outcome in CES patients [7,8].

In addition, the rationale behind the inter patient variation of CES complaints at presentation and the differences in postoperative recovery are not well understood. Some possible factors influencing outcome in CES have been evaluated before, of which time to decompression is the most frequently studied parameter [2,9,10]. The association between MRI and clinical CES features however, has never been studied. Associations between imaging and clinical features were evaluated before for other spinal diseases, such as for spinal lumbar stenosis [11] and for sciatica due to lumbar herniated disc [12]. Identifying MRI characteristics at presentation which are associated with a better or worse outcome of CES after decompressive surgery could substantially improve personalized postoperative care and could lead to a more tailor-made prognosis. Moreover, exploring the relationship between MRI and clinical features at presentation might add to current pathophysiological knowledge, e.g. whether degree of caudal compression at MRI correlates with severity of complaints.

The current study is designed to (1) evaluate the association between MRI features and CES complaints at presentation, (2) evaluate the prognostic value of MRI features for outcome of CES complaints and to (3) compare the lumbar spinal canal diameters of lumbar herniated disc patients with CES, with the diameters of lumbar herniated disc patients without CES (operated because of sciatica) and to the standardized diameters reported in literature.

## Materials and methods

All procedures performed in this study were in accordance with the ethical standards of the institutional and/or national research committee and with the 1964 Helsinki declaration and its later amendments. Approval was granted by the Medical Ethical Committee of the Leiden University Medical Centre (METC), instituted by the Dutch national ethical committee. The IRB waived the need for patient consent and data was accessed anonymously.

In a recent study, the authors described a cohort of 75 patients with CES due to lumbar herniated disc, identified by screening the medical records of all patients operated in the Leiden University Medical Centre (LUMC; university hospital and referral centre for complex spinal surgery) between 1995 and 2010, with the surgery code ‘lumbar discectomy’ or ‘recurrent lumbar discectomy’ (*n* = 744 surgeries) [7]. CES was defined by presence of one or more of the following symptoms: (1) bladder and/or bowel dysfunction, (2) reduced sensation in the saddle area and (3) sexual dysfunction, with possible neurologic deficit in the lower limb. Baseline characteristics and follow up data of identified CES patients were extracted from the medical records. The following items were extracted: gender; age at surgery; duration of complaints of CES at presentation; duration of complaints of herniated disc (defined by presence of sciatica) at presentation; time to decompression (counted from the moment of presentation with CES to first doctor); presence of micturition dysfunction, defecation dysfunction, altered sensation of the saddle area, sciatica (in case it was specified: bilateral or unilateral) and sexual dysfunction, all both at presentation and at two postoperative follow up moments: at discharge from the hospital (follow up moment 1, FU 1) and at check up at the outpatient department two months after surgery (follow up moment 2, FU 2).

For the current study, MRI scans of the lumbar spine of the identified CES patients were retrieved. MRIs had been performed in the LUMC or referring hospitals (Spaarne Gasthuis; Alrijne Hospital; Westfries Gasthuis; Langeland Hospital; Van Weel-Bethesda Hospital) following standardized imaging protocols (synchronized for sciatica study purposes) and were made at the time of presentation, thus prior to surgery. Retrieved MRIs were assessed by an experienced neurosurgeon specialized in spinal diseases, blinded for clinical information of the patient (CVL). The following MRI characteristics were recorded: (1) level of herniated disc, (2) severity of cauda equina compression (mild, moderate, severe) and (3) type of cauda equina compression (unilateral, bilateral: note: median disc lesions were classified as bilateral). No patients with spinal degenerative changes other than herniated disc (e.g. stenosis) were included.

Anteroposterior (AP) diameter of the lumbar spinal canal was measured at mid-sagittal level at MRI in millimetres to the nearest tenth, for each disc level (L1-L2, L2-L3, L3-L4, L4-L5, L5-S1) and each mid-vertebral level (L1, L2, L3, L4, L5). The AP diameter at disc level was measured by drawing a line between the posterior border of the discus and the ligamentum flavum at the midline; for each mid-vertebral level, a line was drawn between the posterior border of the mid-vertebra and the ligamentum flavum. Levels with herniated disc were not measured. AP measurements were only done in MRI scans that were digitally available to maintain high levels of accuracy.

For comparison of AP diameters, the AP diameters of a group of lumbar herniated disc patients without complaints of CES, operated in the same center because of sciatica, were also measured at MRI.

### Statistical analysis

Analyses were done in IBM SPSS Statistics 23.0 (IBM, Armonk, NY, USA). Patient characteristics were analyzed using frequencies. Investigating proportions between unpaired groups of categorical data was done with Chi Square test. Comparison of measurements of the spinal canal between CES patients and lumbar herniated disc patients with sciatica and without CES was done with Mann-Whitney U test.

To evaluate the effect of MRI features on clinical presentation and outcome, binary logistic regression models were built, with MRI features as independent variables (severity of cauda equina compression; type of cauda equina compression i.e. unilateral or bilateral; level of disc lesion) and clinical features as dependent variable. Since there were 4 clinical features (presence of micturition dysfunction, defecation dysfunction, altered sensation of the saddle area and sciatica) measured at 3 different moments (at presentation, FU 1 and FU2), 12 models were created. To correct for possible confounding, the following co-variables were added: gender; age at surgery; duration of CES complaints at presentation; duration of complaints of herniated d**i**sc at presentation. Two extra co-variables were added to the models evaluating clinical features at FU1 and FU2: (1) time to decompression and (2) the evaluated clinical feature at presentation (since dysfunction at presentation is correlated with dysfunction at the next follow up moment). Because of anticipated scarce data on sexual dysfunction, sexual dysfunction was not included in nor analyzed by any regression model. In case of quasi-complete separation of data, the concerning variable was not included in the regression model to maintain high quality analysis.

Prior to running regression models, missing values of the following parameters were handled by multiple imputation with five imputation sets: duration of CES complaints at presentation; duration of sciatica at presentation; time to decompression; defecation dysfunction at presentation, at FU 1 and at FU 2; micturition dysfunction at FU 1 and at FU 2; altered sensation of the saddle area at FU 1 and at FU 2; sciatica at FU 1 and at FU 2. Some numerical data were grouped for analyses, e.g. time to decompression was stratified into six groups: <12 hours, 13–24 hours, 25–36 hours; 37–48 hours; 49–72 hours; >72 hours. Two-sided *p*-values<0.05 were considered statistically significant.

## Results

Due to MRIs that were not available in the archives of LUMC, 27 out of 75 CES patients were excluded. This resulted in a total of 48 included CES patients (Table 1) for whom MRIs were assessed (Table 2).

**Table 1:** Characteristics of CES patients at presentation (n = 48).

	*n*
**Male gender**	22 (45.8%)
**Mean age in years**	42.9 (SD 10.5)
Median duration of complaints of herniated disc in days*	29 (range 1–1095)
Median duration of complaints of CES in hours**	48 (range 1–720)
**Micturition dysfunction**	42 (87.5%)
**Altered sensation of the saddle area**	44 (91.7%)
**Sciatica**	48 (100%)
Unilateral	24
Bilateral	22
Not specified	2
Defecation dysfunction***	28 (70.0%)
Sexual dysfunction****	13 (92.9%)

* available for n = 46

** available for n = 44

*** available for n = 40

**** available for n = 14.

**Table 2:** MRI characteristics at presentation (*n* = 48).

***n*** (%)	
Level of lesion*	
L2-L3	2 (4.1)
L3-L4	4 (8.2)
L4-L5	19 (38.8)
L5-S1	24 (49.0)
Severity of cauda equina compression**	
Mild	10 (22.2)
Moderate	22 (48.9)
Severe	13 (28.9)
Type of cauda equina compression**	
Unilateral	8 (17.8)
Bilateral	37 (82.2)

*the total level of lesions adds up to 49, since one patient had two lesions: at L4-L5 and at L5-S1;

** available for n = 45.

All 48 patients had been surgically decompressed by open discectomy. Timing to decompression was available for 45 patients and was most commonly within 24 hours (*n* = 23) and between 24 to 48 hours (*n* = 14). Three patients were decompressed after 48 hours, but within 72 hours. Five patients underwent decompressive surgery more than 72 hours after presentation to the first doctor with time to decompression of 96 hours (*n* = 2), 120 hours (*n* = 1), 138 hours (*n* = 1) and 216 hours (*n* = 1). Delay was caused by both patient and doctor. Surgery was performed within 24 hours (*n* = 3) and within 48 hours (*n* = 2) after first presentation to the neurosurgeon. Follow up moments took place at two intervals: first follow up moment (FU 1) had a median of 48 hours postoperatively (range 8–336 hours), second follow up moment (FU 2, available for *n* = 34) demonstrated a median of 56 days (4–300 days).

### Association between MRI features and clinical presentation

Thirty-seven patients CES (82%) displayed bilateral compression of the cauda equina at MRI, of whom 19 (51%) indicated that their sciatica was unilateral. There was no correlation between MRI and history of the patient for location of sciatica (*p* = 0.631).

MRI features (severity of cauda equina compression; type of cauda equina compression i.e. unilateral or bilateral; level of disc lesion) were not associated with absence or presence of any of the clinical features (thus micturition dysfunction, defecation dysfunction, altered sensation of the saddle area or sciatica).

A trend was seen for defecation dysfunction at presentation with the co-variable gender, albeit not significant (*p* = 0.061): women more often suffered from defecation dysfunction at presentation.

Note: the MRI parameter ‘type of compression’ (i.e. uni- or bilateral) was removed from the models evaluating micturition at presentation/ FU 1 and altered sensation of the saddle area at FU 2 due to quasi-complete separation (the parameter type of decompression displayed too little variation when analyzing those outcomes). Because all patients suffered from sciatica at presentation, sciatica at presentation was not analyzed as a predictor for outcome.

### Association between MRI features and clinical outcome

MRI features were not demonstrated to be associated with outcome of micturition, defecation, sciatica or altered sensation of the saddle area. The co-variable time to decompression was correlated with sciatica at FU1: a shorter FU time correlated with more sciatica at FU 1 (*p* = 0.043); this correlation disappeared at FU 2.

Note: Sciatica and altered sensation of the saddle area at presentation were removed from the models evaluating clinical outcome of those functions due to quasi-complete separation ((almost) all patients displayed sciatica and altered sensation of the saddle area at presentation).

### Anteroposterior (AP) diameter of the lumbar spinal canal in CES

**Fig 1:**
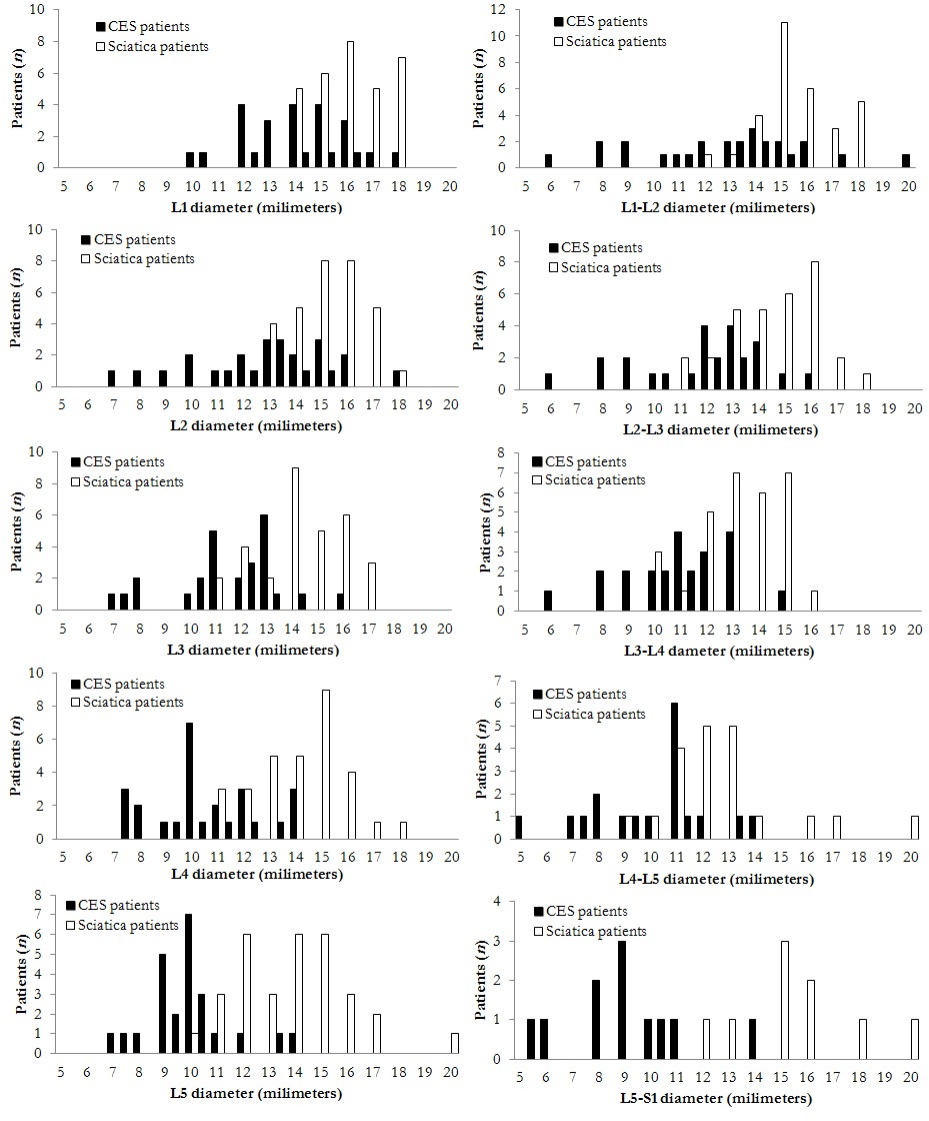
Distribution of the sagittal lumbar spinal canal diameter at vertebral levels (left column) and at disc levels (right column), stratified for CES patients and for sciatica patients.

For 26 CES patients, MRI scans were digitally available and used to measure the AP diameter of the lumbar spinal canal. For comparison, AP diameters of thirty-one patients lumbar herniated disc patients without CES, operated because of sciatica, were measured as well. Patient characteristics known to possibly influence spinal canal size (age, gender) were compared between groups (CES patients with AP measurements; CES patients without AP measurements; lumbar herniated disc patients without CES operated because of sciatica): differences were demonstrated to be non-significant (Table 3). The results of the measurements however, did differ: CES patients displayed a statistically significant smaller lumbar spinal canal diameter at all levels, both disc levels as well as mid-vertebral levels, compared to sciatica patients without CES (largest *p* = 0.002; Table 4; Fig 1).

**Table 3:** Baseline characteristics of CES patients versus sciatica patients.

	CES patients without measurements *n* = 22	CES patients with measurements *n* = 26	Sciatica patients *n* = 31	*p-*value
Male gender (%)	8 (36.4)	14 (53.8)	12 (38.7)	0.396
Mean age in years (SD)	42.3 years (11.2)	43.4 years (10.1)	41.1 (10.6)	0.836

**Table 4:** Measurements of the spinal canal. The mean sagittal diameter of the spinal canal, measured in millimetres to the nearest tenth. Compared between CES patients and herniated disc patients without CES, operated because of sciatica.

	CES patients (*n* = 26)			Sciatica patients (*n* = 31)			*p-*value
	Missing*	Mean ±SD	Min-max	Missing**	Mean ±SD	Min-max	
**L1**	0	14.06±1.99	10.0–18.0	0	16.10±1.40	14.0–18.0	<0.001
**L1-L2**	0	12.92±3.19	6.0–20.0	0	15.58±1.52	12.0–18.0	<0.001
**L2**	0	12.90±2.60	7.0–18.0	0	15.26±1.37	13.0–18.0	<0.001
**L2-L3**	1	11.88±2.40	6.0–16.0	0	14.55±1.77	11.0–18.0	<0.001
**L3**	0	11.54±2.16	7.0–16.0	0	14.32±1.72	11.0–17.0	<0.001
**L3-L4**	3	10.91±2.01	6.0–15.0	1	13.23±1.63	10.0–16.0	<0.001
**L4**	0	10.58±2.02	7.5–14.0	0	14.13±1.77	11.0–18.0	<0.001
**L4-L5**	8	10.06±2.30	5.0–14.0	11	12.75±2.51	9.0–20.0	0.002
**L5**	2	9.94±1.60	7.0–14.0	0	13.87±2.17	10.0–20.0	<0.001
**L5-S1**	15	9.09±2.35	5.5–14.0	22	15.56±2.40	12.0–20.0	<0.001

*not measured due to herniated disc (n = 27, 1 patient had a double lesion); quality too poor at specific level for measurement (n = 2, at L5);

**not measured due to herniated disc (n = 33); quality too poor at specific level for measurement (n = 1, at L5S1).

To compare the measured AP diameters of the CES patients and the sciatica patients with standardized spinal canal diameters reported in literature, studies with a normative distribution of the AP diameter of the lumbar spinal canal, measured at MRI, were searched. Some identified studies were found to be unfit for this comparison since the measured population was biased (e.g. patients referred for low-back pain) [13,14], because the study lacked an exact definition of the subjects for which measurements were taken [15] or because no measurements were available at disc level [16]. The study of Chatha et al. [17] seemed most appropriate for comparison. It describes measurements of the spinal canal in 100 British, symptom-free patients (mean 62 years), who were referred for MRI to screen for presence of metastatic disease, without subsequently having evidence of spinal tumours at the concerning MRI. Even though the study of Chatha et al. is subject to selection bias, the sample size is rather large and patients are quite comparable to the patients in the current study with regard to age, and, in addition, probably quite comparable in terms of race (predominantly Caucasian). In addition, it reports spinal canal size both at intervertebral and disc level, in contrast to aforementioned studies. In order to compare the findings of the current study with the measurements reported by Chatha et al., the average AP spinal canal diameter reported by Chatha et al. was taken as a cut off value. For both the CES patients and the lumbar herniated disc patients without CES, operated because of sciatica, the proportion below the cut off value was indicated (Table 5).

**Table 5:** Proportion with smaller than average diameter. The average sagittal diameters that are used as cut off values are the ones reported by Chatha et al.[17]*.

	% CES patients (*n* = 26)		% Sciatica patients (*n* = 31)		*p-*value
**L1**	53.8		16.1		0.003
**L1-L2**	84.6		54.8		0.016
**L2**	50.0		12.9		0.002
**L2-L3**	96.2		64.5		0.004
**L3**	65.4		16.1		<0.001
**L3-L4**	84.6		51.6		0.001
**L4**	80.8		19.4		<0.001
**L4-L5**	61.5		35.5		0.021
**L5**	88.5		32.3		<0.001
**L5-S1**	38.5		29.0		<0.001

*cut off values (in mm): L1<14.1; L1-L2<15.6; L2<13.2; L2-L3<15.1; L3<12.6; L3-L4<13.8; L4<12.4; L4-L5<12.9; L5<12.4; L5-S1<11.6

## Discussion

As a major finding, this study clearly demonstrates that patients with CES due to lumbar herniated disc have a significant smaller AP lumbar spinal canal diameter than patients with lumbar herniated disc without CES (operated because of sciatica), applying to all mid-vertebral levels as well as disc levels. No associations between MRI features and clinical presentation nor outcome were identified.

Even though the presented cohort is limited, these results may contribute to a beginning of understanding the etiology of CES in herniated disc patients. In addition, the first finding might have potential implications for the selection of lumbar herniated disc patients for decompressive surgery.

### Relation to literature

Spinal canal size of CES patients has not been studied before, however, studies about spinal canal size in patients with other spinal diseases are available. Haig et al. for example, compared patients with low back pain, sciatica and lumbar spinal stenosis with controls, concluding that there is no significant difference between patients and healthy subjects with regard to spinal canal measurements [18].

No associations between MRI and clinical features at presentation nor outcome of CES were identified in this study. Since this is the first study to evaluate this correlation, no references are available to state these results. Similar studies have been performed for other spinal diseases such as sciatica [12] or lumbar spinal stenosis [11,19–21], displaying no correlation between imaging and clinical features, being in line with the current study. The suggestion that factors other than the spinal canal size alone, such as local neurovascular problems, venous obstruction or effect of local inflammatory cytokines–factors that are not identifiable at conventional MRI–contribute to differences in clinical manifestation of CES, seems sensible [18]. Gadolinium-Enhanced MRI may be a promising method in this respect: in stenotic dog models, Kobayashi et al. demonstrated local enhancement at the site of the constricted cauda equina at Gadolinium-Enhanced MRI[22].

A non-significant trend was seen between the co-variable gender and defecation dysfunction at presentation, namely: female gender was associated with more defecation dysfunction at presentation (non-significant: *p* = 0.061). This finding correlates with current literature stating that e.g. constipation is more common in women than in men, in both CES population as well as in the general population [23,24].

The co-variable time to decompression was correlated with sciatica at FU 1: a shorter time to decompression was associated with more sciatica (*p* = 0.0043), which correlation was not demonstrated for FU 2. This finding does not refute the beneficial effects of early decompression which was demonstrated by others [2,9,10,25–28], but rather indicates a correlation between factors indicating a worse prognosis and shorter time to decompression (guided by clinical decision making), such as acute compression of the cauda equina, which is believed to have a worse prognosis than a more gradual compression [25,29].

### Implications

If there truly is a difference in lumbar spinal canal size between lumbar herniated disc patients with CES and lumbar herniated disc patients without CES (operated because of sciatica), this might imply that sciatica patients with a small lumbar canal may need to be approached differently in managing complaints of herniated disc, e.g., it might be beneficial to opt for decompressive surgery earlier in patients with a significant narrow spinal canal size to reduce the risk of developing CES at a later stage. Since this is the only study presently available that evaluated this correlation–and since the setting was retrospective–further prospective research should be conducted before clinical consequences are considered and changes of guidelines are obligatory. A prospective follow up study among sciatica patients would be fitting best, measuring the AP diameters at MRI at presentation—preferably using Gadolinium-Enhanced MRI, also enabling to focus on other factors such as local edema—and ensuring adequate follow up, permitting to correlate the incidence of CES with documented spinal canal size and other MRI features. In case of development of CES, clinical signs and symptoms should be recorded and adequate long term postoperative follow up should take place to evaluate the predictive value of MRI characteristics.

MRI and clinical features were not found to be correlated in the current study. Even though this study has a rather large study population when compared to other CES studies, the limited number of included patients might have caused an inability to detect significant correlations between MRI and clinical features. Aforementioned study proposal with a substantially large cohort and prospective design should be able to give more insights into the predicting value of imaging features in CES patients.

### Limitations

This retrospective study design introduces information bias, e.g. complaints might be reported in the file differently than they were meant by the patient, notes are interpreted differently by the researcher than the clinician originally meant, or notes are simply missing. It is impossible to eradicate this bias completely in the current study design, however, the authors believe bias was minimized by careful assessment of medical notes. Multiple imputation was used to deal with missing values, which was believed to be non-problematic due to the assumption of missing at random. The alternative to multiple imputation would be a complete case analysis, which was believed to be more prone to bias [30].

Potential selection bias is introduced with regard to (1) the included CES patients and (2) the samples of CES and sciatica patients for which AP diameter were measured. Firstly, the inclusion criteria of this study correspond to the most used definition of CES. Indeed the broadness of this definition naturally introduces heterogeneity within the studied population. However, this heterogeneity is inherent to CES and is exemplified by the diversity of clinical manifestations. Division into different groups to create more homogeneity per group (e.g., by using the groups of Tandon and Shankaran [31] or the groups CES-retention and CES-incomplete [32]) would be interesting in a larger cohort, preferably with prospective design. Dealing with the presented cohort size and retrospective study design however, substantial risk of improper grouping and thus low quality analysis lures when dividing included patients into different groups.

Secondly, as displayed in the Results section, CES patients for whom AP diameters were taken form a representative sample of the complete CES cohort and are also similar to the sampled sciatica patients without CES in terms of age and gender, parameters known to influence measurements [11,33,34]. Height was not available retrospectively and therefore not included; however, this parameter was described previously as a possible influencer of spinal canal measurements [35] and therefore, height as a confounder cannot be completely eradicated in the current study.

In this study, no information about degree of decompression is available (i.e. no evaluation of MRI scans was done postoperatively). This could introduce some bias in correlating outcome with the MRI features at presentation: in case decompression was less successful, certainly, more complaints will persist at follow up, which might be not related to the initial MRI features at presentation. However, since all patients were decompressed by similar technique in the same centre, variations in decompression were expected to be minimal.

This study used mid-sagittal AP diameter as indicator of spinal canal size instead of area measurements. AP diameter is proven to be well correlated with area measurements [36] and is currently the measurement most often used in studies relating to spinal canal size. The authors thus believe AP diameter to be a reliable indicator of spinal canal size. The quite recently introduced “reduced interlaminar angle” was proven to be a relevant measurement in the stenotic population in particular, however, was seen as less relevant in the current study population [37].

## Conclusion

There is a difference in lumbar spinal canal size between operated lumbar herniated disc patients with CES and lumbar herniated disc patient without CES (operated because of sciatica). No other MRI characteristics as predictors for presentation or outcome of CES are identified. This finding might imply that sciatica patients with a relative small spinal canal might need to be approached differently in managing complaints of herniated disc, to prevent progression to CES. This hypothesis has to be tested in future studies. Since the current study was retrospective and the number of studied patients relatively small, further prospective research should be conducted before clinical consequences and guideline changes are considered.

## References

[1] Fraser S, Roberts L, Murphy E. Cauda equina syndrome: a literature review of its definition and clinical presentation. Arch Phys Med Rehabil. 2009;90(11): 1964–68. doi: 10.1016/j.apmr.2009.03.021 19887225 10.1016/j.apmr.2009.03.021

[2] Ahn UM, Ahn NU, Buchowski JM, Garrett ES, Sieber AN, Kostuik JP. Cauda equina syndrome secondary to lumbar disc herniation—A meta-analysis of surgical outcomes. Spine. 2000;25(12): 1515–22. 10851100 10.1097/00007632-200006150-00010

[3] Lewis TT. Imaging of the spinal cord and cauda equina. Current Opinion in Neurology and Neurosurgery. 1991;4: 612–6. 10146202

[4] Chang HS, Nakagawa H, Mizuno J. Lumbar herniated disc presenting with cauda equina syndrome. Surg Neurol. 2000;53: 100–5. 10713185 10.1016/s0090-3019(99)00180-9

[5] Jennett WB. A study of 25 cases of compression of the cauda equina by prolapsed intervertebral discs. J Neurol Neurosurg Psychiatry. 1956;19(2): 109–16. 13346384 10.1136/jnnp.19.2.109 PMC497193

[6] Shephard RH. Diagnosis and prognosis of cauda equina syndrome produced by protrusion of lumbar disk. Br Med J. 1959;2(5164): 1434–39. 14445833 10.1136/bmj.2.5164.1434 PMC1991054

[7] Korse NS, Pijpers JA, van Zwet E, Elzevier HW, Vleggeert-Lankamp CL. Cauda equina syndrome: presentation, outcome and predictors with focus on micturition, defecation and sexual dysfunction. Eur Spine J. 2017;26(3): 894–904. doi: 10.1007/s00586-017-4943-8 28102451 10.1007/s00586-017-4943-8

[8] Korse NS, Jacobs WC, Elzevier HW, Vleggeert-Lankamp CL. Complaints of micturition, defecation and sexual function in cauda equina syndrome due to lumbar disk herniation: a systematic review. Eur Spine J.2013;22(5): 1019–29. doi: 10.1007/s00586-012-2601-8 23238848 10.1007/s00586-012-2601-8 PMC3657037

[9] DeLong WB, Polissar N, Neradilek B. Timing of surgery in cauda equina syndrome with urinary retention: meta-analysis of observational studies. J Neurosurg Spine. 2008;8(4): 305–20. doi: 10.3171/SPI/2008/8/4/305 18377315 10.3171/SPI/2008/8/4/305

[10] Todd NV. Cauda equina syndrome: the timing of surgery probably does influence outcome. Br J Neurosurg. 2005;19(4): 301–6. doi: 10.1080/02688690500305324 16455534 10.1080/02688690500305324

[11] Amundsen T, Weber H, Lilleas F, Nordal HJ, Abdelnoor M, Magnaes B. Lumbar spinal stenosis. Clinical and radiologic features. Spine. 1995;20: 1178–86. 7638662 10.1097/00007632-199505150-00013

[12] El Barzouhi A, Verwoerd AJH, Peul WC, Verhagen AP, Lycklama a Nijeholt GJ, van der Kallen BF, et al Prognostic value of magnetic resonance imaging findings in patients with sciatica. J Neurosurg Spine. 2016;24: 978–85. doi: 10.3171/2015.10.SPINE15858 26871651 10.3171/2015.10.SPINE15858

[13] Haig AJ, Weiner JB, Tew J, Quint D, Yamakawa K. The relation among spinal geometry on MRI, paraspinal electromyographic abnormalities, and age in persons referred for electrodiagnostic testing of low back symptoms. Spine 2002;27(17): 1918–25; discussion 1924–5. 12221358 10.1097/00007632-200209010-00019

[14] Wildermuth S, Zanetti M, Duewell S, Schmid MR, Romanowski B, Benini A, et al Lumbar spine: quantitative and qualitative assessment of positional (upright flexion and extension) MR imaging and myelography. Radiology 1998;207(2): 391–8. doi: 10.1148/radiology.207.2.9577486 9577486 10.1148/radiology.207.2.9577486

[15] Pawar I. Kohli S, Dalal V, Kumar V, Narang S, Singhal A. Magnetic resonance imaging in the diagnosis of lumbar canal stenosis in Indian patients. Journal of Orthopedics and Allied Sciences. 2014;2(1): 3–7.

[16] Cheung JP, Samartzis D, Shigematsu H, Cheung KM. Defining clinically relevant values for developmental spinal stenosis. Spine 2014;39(13): 1067–76. doi: 10.1097/BRS.0000000000000335 24732859 10.1097/BRS.0000000000000335

[17] Chatha DS, Schweitzer ME. MRI Criteria of Developmental Lumbar Spinal Stenosis Revisited. Bulletin of the NYU Hospital for Joint Diseases. 2011;69(4): 303–7. 22196386

[18] Haig AJ, Geisser ME, Tong HC, Yamakawa KSJ, Quint DJ, Hoff JT, et al Electromyographic and magnetic resonance imaging to predict lumbar stenosis, low-back pain, and no back symptoms. J Bone Joint Surg Am. 2007;89(2): 358–66. doi: 10.2106/JBJS.E.00704 17272451 10.2106/JBJS.E.00704

[19] Geisser ME, Haig AJ, Tong HC, Yamakawa KSJ, Quint DJ, Hoff JT, et al Spinal canal size and clinical symptoms among persons diagnosed with lumbar spinal stenosis. Clin J Pain. 2007;23(9): 780–5. doi: 10.1097/AJP.0b013e31815349bf 18075405 10.1097/AJP.0b013e31815349bf

[20] Jonsson B, Annertz M, Sjoberg C, Stromqvist B. A prospective and consecutive study of surgically treated lumbar spinal stenosis. Part I: Clinical features related to radiographic findings. Spine. 1997;22(24): 2932–7. 9431629 10.1097/00007632-199712150-00016

[21] Kuittinen P, Sipola P, Saari T, Aalto TJ, Sinikallio S, Savolainen, et al Visually assessed severity of lumbar spinal canal stenosis is paradoxically associated with leg pain and objective walking ability. BMC Musculoskelet Disord. 2014;15: 348 doi: 10.1186/1471-2474-15-348 25319184 10.1186/1471-2474-15-348 PMC4203914

[22] Kobayashi S, Uchida K, Takeno K, Bab H, Suzuki Y, Hayakawa K, et al Imaging of cauda equina edema in lumbar canal stenosis by using gadolinium-enhanced MR imaging: experimental constriction injury. AJNR Am J Neuroradiol. 2006;27(2): 346–53. 16484408 PMC8148809

[23] Johanson JF, Sonnenberg A, Koch TR. Clinical epidemiology of chronic constipation. J Clin Gastroenterol. 1989;11(5): 525–36. 2551954 10.1097/00004836-198910000-00008

[24] Podnar S. Bowel dysfunction in patients with cauda equina lesions. Eur J Neurol. 2006; 13:1112–7. doi: 10.1111/j.1468-1331.2006.01423.x 16987164 10.1111/j.1468-1331.2006.01423.x

[25] Gleave JR, MacFarlane R. Prognosis for recovery of bladder function following lumbar central disc prolapse. Br J Neurosurg. 1990;4(3): 205–9. 2397046 10.3109/02688699008992725

[26] Beculic H, Skomorac R, Jusic A, Alic F, Imamovic M, Mekic-Abazovic A, et al Impact of timing on surgical outcome in patients with cauda equina syndrome caused by lumbar disc herniation. Med Glas (Zenica). 2016;13(2): 136–41. 27452326 10.17392/861-16

[27] Busse JW, Bhandari M, Schnittker JB, Reddy K, Dunlop RB. Delayed presentation of cauda equina syndrome secondary to lumbar disc herniation: functional outcomes and health-related quality of life. CJEM. 2001;3(4): 285–91. 17610771 10.1017/s1481803500005789

[28] Dinning TA, Schaeffer HR. Discogenic compression of the cauda equina: a surgical emergency. Aust N Z J Surg. 1993;63(12): 927–34. 8285904 10.1111/j.1445-2197.1993.tb01721.x

[29] Nascone JW, Lauerman WC, Wiesel SW. Cauda equina syndrome: is it a surgical emergency? University of Pennsylvania Orthopaedic Journal. 1999;12: 73–6.

[30] White IR, Carlin JB. Bias and efficiency of multiple imputation compared with complete-case analysis for missing covariate values. Stat Med. 2010;29(28): 2920–31. doi: 10.1002/sim.3944 20842622 10.1002/sim.3944

[31] Tandon PN, Sankaran B. Cauda equina syndrome due to lumbar disc prolapse. Indian J Orthop. 1967;1: 112–9.

[32] Gleave JRW, Macfarlane R. Cauda equina syndrome: what is the relationship between timing of surgery and outcome? Br J Neurosurg. 2002;16(4): 325–8. 12389883 10.1080/0268869021000032887

[33] Janjua MZ, Muhammad F. Measurements of the normal adult lumbar spinal canal. J Pak Med Assoc. 1989;39(10): 264–8. 2513423

[34] Twomey L, Taylor J. Age changes in the lumbar spinal and intervertebral canals. Paraplegia. 1988;26: 238–49. doi: 10.1038/sc.1988.37 3174169 10.1038/sc.1988.37

[35] Gouzien P, Cazalbou C, Boyer B, Darodes de Taily P, Guenec Y, Sénécail B. Measurements of the normal lumbar spinal canal by computed tomography. Segmental study of L3-L4 and L4-L5 related to the height of the subject. Surg Radiol Anat 1990;12: 143–8. 2396180 10.1007/BF01623341

[36] Gepstein R, Folman Y, Sagiv P, Ben David Y, Hallel T. Does the anteroposterior diameter of the bony spinal canal reflect its size? An anatomical study. Surg Radiol Anat. 1991;13: 289–91. 1803539 10.1007/BF01627760

[37] Kitab SA, Alsulaiman AM, Benzel EC. Anatomic radiological variations in developmental lumbar spinal stenosis: a prospective, control-matched comparative analysis. The Spine Journal. 2014;14: 808–15. doi: 10.1016/j.spinee.2013.09.012 24314904 10.1016/j.spinee.2013.09.012

